# Multifactorial Rare Diseases: Can Uncertainty Analysis Bring Added Value to the Search for Risk Factors and Etiopathogenesis?

**DOI:** 10.3390/medicina57020119

**Published:** 2021-01-28

**Authors:** Domenica Taruscio, Alberto Mantovani

**Affiliations:** 1 National Center for Rare Diseases, Italian National Institute of Health (ISS), 00161 Roma, Italy; 2 Department on Food Safety, Nutrition and Veterinary Public Health, Italian National Institute of Health (ISS), 00161 Roma, Italy; alberto.mantovani@iss.it

**Keywords:** risk analysis, acute inflammatory syndrome, neural tube defects, familial Mediterranean fever, data-poor scenarios, open science

## Abstract

Uncertainty analysis is the process of identifying limitations in knowledge and evaluating their implications for scientific conclusions. Uncertainty analysis is a stable component of risk assessment and is increasingly used in decision making on complex health issues. Uncertainties should be identified in a structured way and prioritized according to their likely impact on the outcome of scientific conclusions. Uncertainty is inherent to the rare diseases (RD) area, where research and healthcare have to cope with knowledge gaps due to the rarity of the conditions; yet a systematic approach toward uncertainties is not usually undertaken. The uncertainty issue is particularly relevant to multifactorial RD, whose etiopathogenesis involves environmental factors and genetic predisposition. Three case studies are presented: the newly recognized acute multisystem inflammatory syndrome in children and adolescents associated with SARS-CoV-2 infection; the assessment of risk factors for neural tube defects; and the genotype–phenotype correlation in familial Mediterranean fever. Each case study proposes the initial identification of the main epistemic and sampling uncertainties and their impacts. Uncertainty analysis in RD may present aspects similar to those encountered when conducting risk assessment in data-poor scenarios; therefore, approaches such as expert knowledge elicitation may be considered. The RD community has a main strength in managing uncertainty, as it proactively develops stakeholder involvement, data sharing and open science. The open science approaches can be profitably integrated by structured uncertainty analysis, especially when dealing with multifactorial RD involving environmental and genetic risk factors.

## 1. Introduction: Characterization of Uncertainties in Risk Assessment and Public Health

Uncertainty analysis is the process of identifying limitations in scientific knowledge and evaluating their implications for scientific conclusions [1]. Currently, uncertainty analysis is a stable component in the process of risk assessment and risk management [1,2,3]; in the meantime, its importance is also envisaged in other different fields relevant to public health, health practice, healthcare and health policy (see, e.g., [4,5]). As a general principle, the scientific experts (the “assessors”) are responsible for characterizing uncertainties, while decision-makers (such as risk managers, health managers, policy-makers) evaluate and decide whether and in what way to take account of the uncertainties. In this way, the identification and assessment of scientific uncertainties becomes an integral component of science-based advice [1,2].

The form and extent of uncertainty analysis may vary widely depending on the nature and context of each issue and the degree of uncertainty that is present. In order to identify uncertainties, the scientific problem should be clearly defined, when possible, in the form of a question (as usually occurs in the field of risk assessment). In particular, it is crucial that the question of interest is well defined, such that the true answer or value could be determined, at least in principle. The specific issues can be widely different, yet must be as clearly defined as possible; in the meantime, uncertainty analyses share some primary questions such as: what is the range of possible answers, and how likely are they? What are the nature and causes of the main sources of uncertainty? Importantly, the latter addresses also what further work is needed to reduce uncertainty. There is no “true and absolute” uncertainty: uncertainty analysis has to express the uncertainty of the “assessors” at the time they conduct their assessment [2].

As a general approach, uncertainties should be:

(i) Identified in a structured way to minimize the chance of overlooking relevant uncertainties;
(ii) Prioritized on the basis of their likely impact on the outcome of scientific conclusions.

When dealing with complex issues, it may be important to characterize uncertainties separately for parts of the questions; for instance, in the risk assessment of toxic contaminants, a separate analysis can be performed for uncertainties related to exposure (e.g., validity of chemical analytical methods) and to effects (e.g., extrapolation of toxic effects from experimental animals to humans) (see [3] for a detailed discussion). However, it is also necessary to characterize the global burden of uncertainty in the scientific conclusions; as far as it is possible, the overall impact of uncertainties should be expressed in a semi-quantitative way, e.g., slight, moderate, heavy. The assessment of the impact is important in order to provide a correct, transparent, understandable and usable message to those roles (e.g., risk managers, healthcare providers, clinicians) that will utilize the scientific conclusions. Finally, prioritizing uncertainties for future investigation will support recommendations for data collection and/or research.

The sources of uncertainty may be highly diverse and can be generally clustered into great groups [4]:

- Epistemic uncertainties, which are directly related to lack of knowledge;
- Sampling uncertainties (also called “stochasticity”), associated with the available data and related to inherent randomness;
- Uncertainties related to natural variability.

The boundaries between sampling uncertainty and uncertainties due to natural variability may not always be readily evident, yet they should be kept conceptually distinct. For instance, the sampling uncertainty is increased by the insufficient precision of a test method (hence being vulnerable to random error); a different sort of uncertainty is due, e.g., to the natural variability due to the frequency of genetic mutations associated with disease risks in different populations [6].

As a general view, uncertainty is related to knowledge and sampling, while variability refers to actual heterogeneity in the real world; thus, contrary to uncertainty, variability cannot be altered by obtaining more information because it refers to real differences. However, natural variation may produce uncertainties when knowledge of the variability for the relevant parameter(s) in a defined population is incomplete (as it is often the case); moreover, variability can lead to difficulties in synthesizing information from disparate sources. In order to deal with variability in scientific assessment, a primary requirement is to clearly identify the population involved, as well as any relevant sub-populations: for instance, the levels of folate in red blood cells of women of fertile age living in a defined geographic area, and within this population, the sub-population taking folate supplements [7]. If the population or individual values change over time, it is also necessary to specify the time period of interest [1,2,4].

All three great groups of uncertainties have some overlaps and eventually lead to limitations of knowledge, but the approaches to mitigate them are somehow different. Epistemic uncertainties are directly amenable through a clear definition of research needs, while the other kinds of uncertainties are, to some extent, unavoidable, yet they have to be recognized and managed. These considerations further highlight the need for systematic assessments of the factors associated with uncertainties across public and healthcare areas. Table 1 shows the definitions for key entities discussed in this paper.

Our paper aims at starting a discussion on the specific aspects of uncertainty analysis in the field of rare diseases, with particular attention paid to conditions with multifactorial (gene–environment or gene–gene) origins.

## 2. Uncertainty Analysis and Rare Diseases

The uncertainty issue is inherent to RD. Indeed, a recent paper recognized the relevance of uncertainty analysis in regard to treatments for RD and proposed a tool, TRUST4RD (Tool for Reducing Uncertainties in the evidence generation for Specialised Treatments for Rare Diseases), to identify, review and prioritize uncertainties for decision-makers by developing an iterative and informed dialogue amongst stakeholders. The background for developing TRUST4RD is that approval of treatments for RD is often based on small or uncontrolled trials; indeed, trials of sufficient size are often difficult to conduct, or repeat, due the rarity of the condition, sparsity of patients and/or ethical reasons. By defining uncertainties in the assessment of value and value for money of RD treatments, the tool aims at strengthening the weight of evidence supporting the discussions on the authorization of specific treatments [5].

TRUST4RD is, therefore, specially tailored to the selection of treatments. A similar effort for a systematic approach toward uncertainty analysis is not currently implemented in regard to other major areas in the RD field, namely, identification of risk factors, etiopathogenesis and diagnosis. Nevertheless, these critical areas are affected by major sources of uncertainties, such as the rarity of the condition and the difficult collection of cases [8].

This scenario brings a burden made up by epistemic and sampling uncertainties as well as by population variability; the resulting impact on prevention and/or diagnosis may be far-reaching. For instance, primary prevention of rare and severe birth defects, such as holoprosencephaly, is still difficult because of the knowledge gaps in modifiable (environmental exposures, diet and lifestyles) risk factors. In this respect, two different types of evidence should be integrated, namely, on epidemiological associations and on biological plausibility [9,10].

Due to epistemic and sampling uncertainties, datasets may highlight different clinical aspects while failing to identify a common pathogenesis, especially in the case of newly recognized disease entities [11]. The relevance of uncertainties in designing a consistent clinical entity is highlighted in such RD as familial Mediterranean fever (FMF) [12]. In these cases, uncertainties may result in misdiagnosis with serious consequences. Abdominal attacks of FMF may simulate acute appendicitis; the similar presentation of the two clinical entities often leads to an unnecessary appendectomy [13]. Overall, prevention and diagnosis of RD often face the challenges posed by the need for competent management with an insufficient knowledge base and/or diagnostic capability: a kind of scenario that may parallel those faced by risk assessors and risk managers in data-poor situations of environmental and/or food chain pollution [14].

It is interesting to view how a major risk assessment body, the European Food Safety Authority (EFSA), has dealt with uncertainties in regard to the pathogenesis of a rare condition, infant leukemia, and to its environmental risk factors [15].

Infant leukemia occurs in <1-year-old infants and recognizes an in utero origin at an early phase of fetal development. Rearrangements of the mixed-lineage leukemia (MLL) gene producing abnormal fusion proteins are the most frequent genetic/molecular findings in infant B cell acute lymphoblastic leukemia. In small epidemiological studies, mother/fetus exposures to pesticides have been associated with infant leukemia; while the strength of evidence and power of these studies were weak, the evidence was consistent enough as to trigger an assessment of the biological plausibility of such association by the EFSA. Experimental in vitro or in vivo models do not sufficiently recapitulate the human disease and regulatory toxicology studies—customarily performed on pesticides—are unlikely to capture a specific tumorigenic hazard originating in utero. Therefore, the EFSA developed an adverse outcome pathway (AOP) approach. The AOP approach condenses molecular, pathological, toxicological and clinical knowledge in a pragmatic, transparent and weight of evidence-based framework: in each AOP, early molecular events are connected by necessary steps (key events) leading to an adverse outcome, which can be a clinical entity; AOP might also be considered as a stepwise and standardized description of “pathogenesis”. When the mechanisms and molecular and cellular effects of a chemical (or another risk factor) fit into a certain AOP, this may be considered as evidence that the chemical (or risk factor) is linked to the relevant adverse outcome [16]. The EFSA has substantially based the AOP for infant leukemia on an analogous disease—secondary acute leukemia caused by the topoisomerase II (topo II) poison etoposide—and on cellular and animal models [17]. The hallmark of the AOP is the formation of MLL gene rearrangements via topo II poisoning, leading to fusion genes and ultimately acute leukemia by global (epi)genetic dysregulation: a big “hit” in utero on MLL is currently identified as the single essential key event. While the EFSA opinion pointed out that pesticide chlorpyrifos, and possibly similar compounds, can induce key molecular and cellular events relevant to this AOP, we wish to highlight the identification of uncertainties about etiology and risk assessment, including the specific embryonic target cell during the short and spatially restricted period of susceptibility, and the role of (epi)genetic features modifying the initiation and progression of the disease [15,17].

First, a prerequisite for the specific outcome, i.e., creation of chromosomal rearrangement, is that topo II inhibition has to occur in an especially vulnerable and correct hot spot in the MLL locus; however, details of this process and how it happens are not clear. In addition, the potential role of other reciprocal fusion genes has not been studied.

While hematopoietic stem cells in fetal liver are plausible suspects, a leukemia-initiating cell has not been identified with sufficient confidence; consequently, there is no target cell model to recapitulate the linkage between topo II inhibition (“poisoning”) and the production of double-strand breaks (DSB) in an appropriate target.

Overall, the empirical support provided by in vivo experiments is limited and also the dose–response relationships between etoposide (the foremost model chemical) and treatment-related leukemia are difficult to unra)vel. MLL-AF4(ALL1-fused gene from chromosome 4) in frame fusion is a rare event that needs to occur in a target cell within a relatively small and spatially restricted cell population during the appropriate, epigenetically plastic developmental window; thus, it may be difficult to empirically support this process. The difficulty in modeling the cellular concentration of etoposide (or an active metabolite) in vivo lies both in gaps of knowledge on kinetics (epistemic uncertainty) and in an inherent and unavoidable difficulty: the concentration resulting in a proper fusion gene should be in a relatively narrow range, high enough to lead to a partially repaired insult yet low enough to avoid cell death and allow cells to accumulate the abnormality.

The AOP, currently based on the MLL “hit” in utero as the single essential key event, might be actually more complex. The activation of cellular proliferation by mutation or other (epi)genetic insults might be necessary for overt leukemia. Thus, a significant epistemic uncertainty is about what events (if any) are essential to convey a proliferative advantage to cells with MLL translocation.

Overall, in utero evidence of the disease is difficult to obtain in humans and one has to resort to in vitro cellular systems, which may be inadequate to take into consideration the potential effects of tissue microenvironments, rapidly changing developmental stages and also the possible role of (yet unidentified) factors facilitating the proliferation of cells with MLL translocation. Animal models are available, but they are also a source of uncertainty due to species-specific features. For instance, MLL-AF4 knock-in mice develop leukemia only after a prolonged latency, thus not recapitulating the “pathognomonic” feature of infant leukemia. A related important uncertainty derives from toxicological testing. A clear understanding of a higher sensitivity to certain chemicals of fetal vs. mature hematopoietic cells is lacking, particularly because fetal hematopoietic stem cells are not present in the standard genotoxicity test battery for chemicals. More chemicals and comparative assays should be tested to scientifically validate this cell system.

Finally, such as for all RD with environmental risk factors (see also the following case study on neural tube defects), an important source of uncertainty is to provide a convincing and evidence-based explanation for the dilemma concerning the rarity of disease in the face of pervasive exposure to “environmental” topo II inhibitors (including organophosphorus pesticides). It is also demanding to design epidemiological studies powerful enough to provide robust answers [15,17].

In the following sections, we will briefly discuss three case studies, represented by multifactorial (gene–environment or gene–gene origin) RD, namely:

- Acute multisystem inflammatory syndrome in children and adolescents associated with SARS-CoV-2 infection (main question: definition of the disease);
- Neural tube defects (main question: assessment of the risk factors and protective factors);
- Familial Mediterranean fever (genotype–phenotype correlation).

The case studies are intended as examples to highlight the relevance of uncertainty analysis in the characterization of risk factors and etiopathogenesis of RD. While a systematic analysis of the literature is beyond the scope of this paper, the evidence discussed is selected on the basis of expert judgement in order to identify examples of issues that may significantly impact on RD prevention and/or diagnosis. Table 2 summarizes the questions, types and flow of information and impacts of uncertainties for the three case studies.

### 2.1. Case Study 1. Definition of the Disease: Acute Multisystem Inflammatory Syndrome in Children and Adolescents Associated with SARS-CoV-2 Infection

The available scientific evidence indicates that the clinical course of SARS-CoV-2 infection occurring in pediatric patients has low lethality rates; however, a number of scientific publications from different world areas describe a novel acute multisystem inflammatory syndrome in children and adolescents, associated with positivity for SARS-CoV-2 or with the presence of antibodies that are anti-SARS-CoV-2. This syndrome shares some clinical features with Kawasaki disease (KD), a rare systemic vasculitis of small and medium-sized vessels, that mainly affects children aged between 1 and 5 years. Shared features include an aberrant inflammatory response and some therapeutic options (immunoglobulins, steroids, anti-cytokine drugs). The new syndrome, however, differs from KD by a number of other characteristics, such as the older age of the affected subjects (<21 years), the severe multisystemic involvement, including myocardial and/or gastrointestinal involvement, and the plausible correlation between SARS-CoV-2 infection and the onset of the syndrome [18].

Different national and international scientific bodies have identified this previously unrecognized disease by different names as well as by different diagnostic criteria. Following also the initial debate on the distinction between the new syndrome and the well-recognized KD (which bears no relationship to SARS-CoV-2), a consistent clinical picture is emerging, which prompts the adoption of clinical diagnostic criteria. Most patients with the new syndrome have antibodies against SARS-CoV-2, and the virus is detected in a smaller proportion. However, no causal explanation of such relationship is available as yet [11,18]. A study on patients pointed out changes in immune cells indicative of impaired antigen presentation, which might provide a clue toward pathogenesis [19]. Another recent study analyzed the inflammatory response in the syndrome: this differs from the cytokine storm of severe acute COVID-19, as well as from the features of KD, based on T cell subsets involved, interleukin-17A and biomarkers associated with arterial damage. The authors also pointed out that the relevance of T cell differences between KD and the new syndrome is uncertain because the two sets of patients show differences in age [11,20].

Acute multisystem inflammatory syndrome is uncommon (2 in 100,000 persons <21 years of age) as compared with SARS-CoV-2 infection diagnosed in persons younger than 21 years of age over the same period (322 in 100,000) [21]; a number of studies also point out a differential risk related to ethnicity. Rarity and ethnicity hint to the intervention of other factors, which are currently undefined [11]. Last but not least, as M. Levin pointed out [11], children meeting current diagnostic criteria for the new syndrome might just be the “tip of the iceberg” of a bigger problem involving forms of unexplained multi-organ acute inflammation in children and adolescents. Based on similarities of the immunological disruption patterns, the pathways leading to acute rheumatic fever and toxic shock syndrome have been recently indicated as potential clues to the pathogenesis of the new acute multisystem inflammatory syndrome [22]. Thus, the uncertainty in the syndrome’s etiopathogenesis bears an obvious, direct relationship with the definition of the clinical spectrum and hence of criteria for diagnosis and epidemiological surveillance.

Overall, the main epistemic uncertainties reside in the definition of the causal link with SARS-CoV-2 as well as in the involvement of risk-modifying factors which—albeit likely—remain undefined. As a direct consequence, the range of clinical phenotypes and the diagnostic criteria remain incompletely defined.

The main sampling uncertainties reside in the varying levels of awareness and attention toward acute multisystem inflammatory syndrome in children and adolescents, with an obvious impact on the accuracy of the syndrome’s epidemiology. Finally, both kinds of uncertainties are increased, to an unknown extent, by the inherent variability of the populations at risk in terms of potentially relevant traits, such as ethnicity, genetic polymorphisms or even environmental factors.

The impact of uncertainties is heavy, as it does directly affect healthcare, in terms of both primary/secondary prevention and diagnosis and, consequently, epidemiology and clinical management. The uncertainties in the pathogenesis and clinical manifestations impact on treatment decisions about preventing a progression to shock and multiorgan failure; it is also uncertain whether children with self-resolving inflammation might have sequelae requiring a longer-term follow-up [11]. For instance, whereas hepatitis is observed and is associated with a more severe presentation, knowledge about the long-term impact on the liver, if any, is insufficient [23]. Overall, the case of acute multisystem inflammatory syndrome well represents the relevance of uncertainties when a new, rare clinical entity is identified.

### 2.2. Case Study 2. Assessing Risk Factors and Protective Factors: Neural Tube Defects

Neural tube defects (NTDs) are congenital anomalies due to the improper closure of the neural tube, the main ones being anencephaly, encephalocele and spina bifida. Overall, NTDs feature among the most important congenital anomalies, both because of their incidence and their clinical consequences. The total (live births plus fetal deaths after 20 weeks of gestational age plus terminations of pregnancy for fetal anomaly) incidence in Europe has been estimated in the order of 9 per 10,000; the live birth incidence is lower, especially for the most severe NTDs such as anencephaly [24]. Growing, albeit still limited, evidence indicates that the incidence may be significantly higher in low- and middle-income countries [25]. While the prognosis is very variable and mainly depends on the tract of the neural tube affected, NTDs invariably lead to, at least, some degree of disability: the most severe one, anencephaly, always causes perinatal death.

NTDs are typical multifactorial events involving both genetic and environmental factors [26]. A systematic approach toward NTD pathogenesis has been proposed only recently, through the identification of likely targets: proteins and protein interactions involved in neural tube patterning and morphogenesis, as well as signaling pathways such as the retinoid pathway [27]. Nevertheless, this group of congenital anomalies is a telling example of evidence-based, feasible and affordable primary prevention [28,29]: in fact, increasing the folate status of the mother in the periconceptional phase up to the end of human embryonic organogenesis can bring a significant reduction in NTD incidence, in the range of 30–50% and up to 70%, especially in countries with higher incidence ([28,30], and references therein). While there is general agreement about the value of a vegetable-rich and balanced diet and of periconceptional supplementation with folic acid (the synthetic and most stable form of the vitamin folate), there is still debate about folic acid fortification of flours [30]. Some authors consider flour fortification as the major strategy for primary prevention of NTDs, provided that levels of unmetabolized folic acid are monitored periodically in the population [30]; meanwhile, a considerable uncertainty remains on whether and at what intake level folic acid (a strong epigenetic modulator) may act as a tumor promoter, particularly in regard to colorectal cancer [31]. Waiting for a robust, science-based risk-to-benefit assessment supported by a thorough uncertainty analysis, different perceptions and viewpoints have led to different approaches: folic acid fortification is supported in a number of countries such as the USA and Israel but is not supported in the European Union where higher food safety standards are endorsed. Surely, the risk-to-benefit assessment of folic acid fortification is still an open scientific issue; attempts to deny the significant uncertainties needing to be addressed (as, e.g., in [32]) just result in a hindrance to the progress of public health and primary prevention. Useful overviews of the complex interactions (and related uncertainties) among dietary folate intake, supplementation and/or fortification with folic acid, genetic susceptibility and NTD are provided by [30,33].

Maintaining the focus solely on folate status and folic acid intake would overlook a relevant question: what are the risk factors for folate-unresponsive NTDs that can reach up to 50% or more of the overall incidence? While some cases are due to teratogenic drugs, mainly the antiepileptic valproate [34], it is highly plausible that a main component is due to environmental factors interacting with genetic susceptibility. This hypothesis builds upon the scenario of “widespread exposure leading to the adverse outcome in a few susceptible individuals”, postulated also for childhood leukemia [15,17] and other congenital anomalies [10,11]. Another related question is whether a putative environmental factor antagonizes the action of folate and/or acts through a folate-independent mechanism.

The use of an AOP may help in this respect. The official AOP repository, the Collaborative Adverse Outcome Pathway Wiki (AOP-Wiki, https://aopwiki.org), includes AOP 275: “Histone deacetylase inhibition leads to neural tube defects” [35]. The expression and function of histone deacetylases (HDACs) play a pivotal role in the development of the nervous system. HDAC inhibition during the first weeks of neurodevelopment, before or around the time point of neural tube closure, may lead to an imbalance of histone modifications and eventually to altered gene expression and differentiation of neuroectodermal cells that cannot close the neural tube anymore. The identification of main target genes for NTD will help in refining the AOP [27]. HDAC inhibition is the putative mechanism underlying valproate-induced NTD [36] and has no direct connection with folate metabolism.

The relevant question for NTD primary prevention is, then, whether widespread contaminants can reach the embryonic neural tube and act as HDAC inhibitors. One example is the mycotoxin fumonisin B1 (a contaminant of grains): fumonisin B1 induces folate-independent NTD in mice, related to HDAC inhibition [37,38]. Some epidemiological evidence of an association between maternal fumonisin exposure and NTD in low-income populations already exists [39]; studies in high-income areas, such as Europe, may be warranted because the presence of the mycotoxin is expected to increase along with climate changes [40]. The fumonisin case may be taken as a proof of principle that widespread contaminants can act as HDAC inhibitors in embryonic tissues.

Another potential mechanism, still not described as an AOP, is related to the inositol status of the maternal embryonic unit; an altered inositol status might be triggered by diabetes with its related nutritional, metabolic and environmental risk factors [41]. Indeed, risk genes for myelomeningocele (an NTD involving the spinal cord) pertain to the folate/one-carbon metabolism or to the glucose homeostasis/oxidative stress networks [42]. Other environmental risk factors for NTD are reported in the literature, such as high levels of Cesium-137 [43]; however, the available evidence does not allow for concluding whether these act through folate-responsive or folate-independent pathways.

Concerning the main question of effective primary prevention of NTDs, the main epistemic uncertainties include the risk-to-benefit analysis of flour fortification with folic acid and the identification of environmental risk factors for folate-unresponsive NTDs, based on the relevance of mechanisms and on the ability to reach the embryonic compartment.

The main sampling uncertainties pertain to inaccuracies and discrepancies in monitoring the NTD epidemiology and the relevant biomarkers, e.g., folate status. Both kinds of uncertainties interact with relevant aspects of population variability, related to socio-economic status, ethnicity, diet and genetic polymorphisms.

The overall impact may be moderate on the potential for primary prevention because an adequate folate status is already established as a factor reducing, by approximately 50%, NTD incidence. However, the impact of both epistemic and sampling uncertainties may be severe on the ability to follow up and assess the effects of primary prevention measures. Indeed, a study carried out in the USA recently reported an analysis of a food fortification dataset, highlighting better socio-economic status as a stronger protective factor compared to folic acid fortification [44]. The identification of uncertainties, therefore, indicates comparable and quality-controlled data on the benefits of protective factors and preventive actions as a priority need for public health action towards NTDs.

### 2.3. Case Study 3. Genotype–Phenotype Correlation: Familial Mediterranean Fever

Familial Mediterranean fever (FMF) is a monogenic autoinflammatory disease with worldwide distribution. The disease is caused by mutations in the Mediterranean Fever—innate immuity regulator (MEFV) gene encoding the inflammasome sensor pyrin and is significantly more frequent in populations of the Mediterranean area. The phenotype is characterized by attacks of painful periodic fever with diffuse serositis and risk of secondary amyloidosis. The disease appears to be transmitted through autosomal recessive mutations, with about 300 variants reported worldwide. However, their association with symptom severity, the relative frequencies of variants in different populations and the disease penetrance are far from being completely understood [45]. Clinical diagnosis of FMF is complicated by an overlap in symptoms with other diseases, and interpretation of genetic testing is confounded by the lack of a clear genotype–phenotype correlation together with a full understanding of pathogenetic mechanisms. According to a systematic review by Gangemi et al. [46], the *p.M694V* mutation was reported to have a relatively severe clinical course; similarly, patients homozygous for M694I and M680I, or carrying a combination of both at codons 694 and 680, have a severe disease. Further, patients homozygous for M694V and V726A variants experienced a more severe clinical picture. Conversely, heterozygous *p.V726A* and *p.E148Q* genotypes have been correlated with a milder disease course. At present, doubts remain on the potential pathogenic role of the E148Q variant. Overall, the heterogeneity in clinical FMF manifestations reflects the changes occurring in the repertoire of mutations. Genotype–phenotype relationships are also important to characterize specific clinical aspects of FMF, such as neurological manifestations [47].

A further uncertainty concerns the model of inheritance. In a study on 107 Italian patients [48], nine distinct mutations were detected, with 85.98% of patients showing a heterozygous genotype. While the most common genotypes were *p.Met680Ile/wt* and *p.Met694Val/wt*, no significant difference in clinical phenotype was observed among heterozygous, homozygous and compound homozygous subjects; as data supporting that, contrary to a recessive autosomal inheritance model, heterozygous patients fulfilled the criteria of clinical FMF. Two variants, *p.Met694Val/wt* and *p.Met680Ile/wt*, were associated with the most severe clinical phenotype; eight variants (*p.Ala744Ser/wt*, *p.Glu148Gln/Met680Ile*, *p.Met680Ile/Met680Ile*, *p.Met680Ile/Met694Val*, *p.Pro369Ser/wt*, *p.Met694Ile/wt*, *p.Glu148Gln/Glu148Gln*, *p.Lys695Arg/wt*) resulted in 100% pathogenicity. Overall, the authors considered that the model of inheritance might be a “non-classic” autosomal recessive inheritance as well as an “atypical” dominant autosomal inheritance with incomplete penetrance and variable expressivity.

In a recent large study on 1028 FMF patients carried out in Turkey, the most common genotypes were M694V/R202Q heterozygous (10.4%) and M694V homozygous (7.5%), all other genotypes occurring at rates below 5%; new variants were also described. In addition, the study investigated whether the MEFV mutations were exon 10 or non-exon 10, by clustering the patients in four groups: group 3 (exon 10 homozygous or compound heterozygous) was correlated with a higher risk of appendectomy, while most other symptoms showed no significant correlations with the clusters. The authors recommended to analyze all exons in the MEFV gene with next-generation sequence analysis in the detection of FMF disease [49].

Since the conventional autosomal recessive inheritance model does not fit FMF, Stella et al. [45], using in silico tools, demonstrated a significant association of variants’ pathogenicity with their position along the coding sequence but not with variants’ frequency.

The current uncertainty in the genotype–phenotype correlation for FMF also includes the contribution of modulating environmental factors, particularly those related to the diet and its influence on the microbiome [50]. Indications on specific diet- and microbiome-related risk factors from the recent literature include high systemic concentrations of short-chain fatty acids [51] and high wheat consumption [52].

In regard to the question on genotype–phenotype correlations in FMF, the epistemic uncertainties concern the pathogenic role of the numerous variants concerning the severity of the syndrome and specific clinical manifestations, the “non-classical” model of inheritance and the modifying role of environmental (dietary) factors.

The sampling uncertainties concern the potential for an accurate diagnosis and, in particular, for a characterization of genetic variants. Both uncertainties interact with the variability in the populations concerning the distribution of genetic variants and, possibly, also dietary habits.

The overall impact of uncertainties is null as regards the etiopathogenesis, which is well defined, but is moderate to severe concerning the secondary and tertiary preventions of the disease manifestations and also the diagnosis and epidemiology. The uncertainties in the causative role of variants identified in mutation screening specifically affect the accuracy of genetic counselling and prognosis [45]. Uncertainty analysis pinpoints priorities: new diagnostic tests that can tackle functional subtyping [53] as well as the modulating role of dietary components [51,52]. Therefore, the FMF example illustrates how also conditions identified as “monogenic” can show uncertainties with a significant impact on healthcare.

## 3. Discussion

The broad field of RD is widely recognized to be prone to knowledge gaps that ultimately result in hindrances to prevention, diagnosis, treatment and healthcare. The many sources of knowledge gaps include, yet are not limited to: the wide number and diversity of RD; the difficulties in performing epidemiological studies and randomized control trials, related to the paucity and sparsity of cases; and the limited economic stimulus in the research and development of new tools for those conditions involving globally just hundreds or few thousands of patients [54,55].

A sector highly vulnerable to knowledge gaps is represented by multifactorial RD, which may include rare tumors, congenital anomalies and inflammatory conditions [11,17,28,29]. A major, specific uncertainty consists in establishing a link between widespread risk factors and rare adverse outcomes, as outlined, e.g., for infant leukemia and pesticide exposure [17] as well as for the inflammatory syndrome in children and adolescents associated with SARS-CoV-2 infection [11]. The systematic analysis of uncertainties, along the model developed in the field of risk assessment [1,2], may be an important tool for the RD community in order to describe, assess and reduce the negative impact of knowledge gaps. In particular, the analysis of epistemic uncertainties identifies the research needs as well as supporting their prioritization, while the sampling uncertainties may pinpoint the need for harmonization and quality assurance of diagnostic and epidemiological tools.

Uncertainty analysis in RD may present aspects similar to those encountered when conducting risk assessment in data-poor scenarios, where there is the need to address the action of risk managers, and often urgently so, with largely insufficient datasets [14]. In this respect, the expert knowledge elicitation (EKE) approach may be considered with interest in the field of RD. EKE requires the availability of a group of experts, independent and with different backgrounds, and has to be well managed by somebody proficient in the process. Upon these conditions, EKE is an efficient, robust and standardized tool in the area of risk analysis and public health in order to describe uncertainties and their distribution and range [56,57]. Especially in data-poor scenarios, EKE may also facilitate a reduction in the range of uncertainties, by promoting consensus through discussion. It could be, therefore, useful to explore the consistent use of EKE-like procedures within expert groups dealing with RD.

The case studies presented show both the relevance of uncertainties for tailoring specific health interventions, as well as the need for designing uncertainty analysis in RD as a multi-step process.

Without any ambition of systematic analysis (definitely beyond the scope), the selected cases outline the potential to identify epistemic and sampling uncertainties and their overall impact on defined questions relevant to health interventions: definition of an emerging syndrome, factors for primary prevention, factors influencing genotype–phenotype correlations. Pending the clear definition of the question, identification of uncertainties could be undertaken as a default step. A screening step would be needed in order to identify the relevant one. Uncertainties are identified as “relevant” when they have the potential to impact, significantly, on decision making in regard to prevention and/or diagnosis and/or healthcare. The characterization—i.e., identification and prioritization—of relevant uncertainties requires expert judgement, possibly involving EKE approaches. One outcome of unambiguous characterization of uncertainties is pointing out the priority issues for further investigation. In the FMF case study, the etiopathogenesis is known: the research priority is the pathways by which genetic variants and environmental factors modulate the syndrome’s phenotype. In its turn, this uncertainty has a negative impact on genetic counselling, diagnosis and prognosis [45]. As for the NTD case study, the role of an adequate folate status as a protective factor is established; however, the epistemic uncertainties (environmental risk factors, full safety of the folic acid fortification) and the sampling uncertainties (epidemiology of NTDs, measurements of folate status) may have a serious impact on the ability to assess the effects of primary prevention (see e.g., [44]).

A structured and detailed analysis of uncertainties may be liable to a misperception. In principle, healthcare providers at all levels, health policy-makers and/or risk managers expect—understandably—to receive straightforward answers; therefore, highlighting uncertainties may be perceived as in contrast with providing scientific bases for action. Actually, uncertainty analysis increases the robustness and transparency of scientific assessment underlying the public health decisions: it both describes the available scientific evidence and, by indicating the areas where uncertainties exert the main impacts, also indicates the priority topics for strengthening the evidence. Indeed, several authors from different and unrelated public health areas have discussed and highlighted the importance of uncertainty analysis to support the robustness of scientific evidence and its translation into action [58,59,60,61].

Finally, the RD area offers a main strength for managing uncertainty: indeed, the RD area proactively develops stakeholder involvement, data sharing and open science [5,49]. The expanded role of patient advocacy organizations and patient engagement continues to gain acceptance within the research community, making a case for open science implementation; future developments envisage a greater understanding of available information from multiple sources including electronic health records and big data sources [55,62]. Open science, founded on wide sharing of data and knowledge, is a value deeply felt in the RD community, in order to cope with the several, well-recognized limitations that are inherent to RD, such as insufficient patient data and resources [49]. An important effort toward a road map for open science in RD is ongoing, which includes—among others—tools that enable patients to share their own data, standards for consistent representation of phenotype data, optimizing interoperability of registries and networks of controlled-access data that can be searched using diagnostic algorithms [61].

## 4. Conclusions

Uncertainty analysis in RD prevention and diagnosis is important and timely. We propose that the open science approaches developed by the RD community can be profitably integrated by structured models for uncertainty analysis in order to describe the impact of uncertainties and prioritize further research. We also suggest that uncertainty analysis may be especially relevant when dealing with multifactorial RD involving different genetic and non-genetic risk factors.

## Figures and Tables

**Table 1:** Definitions for key entities relevant to uncertainty analysis of risk factors and etiopathogenesis in multifactorial rare diseases.

Uncertainty	Limitation in Scientific Knowledge That May Impact on Scientific Conclusions Relevant to Risk Analysis, Public health, Health Practice, Healthcare and Health Policy [1,2,3,4,5]
Epistemic uncertainty	Uncertainty directly related to lack of knowledge; it is reduced by properly addressed further studies [4]
Sampling uncertainty	Uncertainty associated with the available data and related to inherent randomness; it can be reduced by increasing precision and/or harmonization of methods [4]
Variability	The unavoidable heterogeneity within a population; if not properly characterized, it can be a source of uncertainty [4,6]
Impact of uncertainty	The extent by which uncertainties, either individually or as a group, affect the robustness of scientific conclusions: it is usually expressed in a semi-quantitative way, e.g., slight, moderate, severe [1,2,3]
Expert Knowledge Elicitation	A guided and iterative process to exploit the expertise and experience of a group of experts in order to describe uncertainties [2]

**Table 2:** Overview of questions, types and flow of information and impacts of uncertainties for the three case studies: acute multisystem inflammatory syndrome in children and adolescents associated with SARS-CoV-2 infection; neural tube defects; familial Mediterreanean fever.

Condition	Question(s)	Epistemic Uncertainties	Sampling Uncertainties	Impact(s)
Acute multisystem inflammatory syndrome in children associated with SARS-CoV-2	Causal link with SARS-CoV-2 Possible risk-modifying factors	Model explaining pathogenesis Range of clinical phenotypes Diagnostic criteria	Varying levels of awareness and attention toward the syndrome Effect of potentially relevant traits (e.g., ethnicity, genetic polymorphisms)	Severe (both epistemic and sampling uncertainties), on all aspects of healthcare: primary/secondary prevention, diagnosis, epidemiology clinical management
Neural Tube Defects (NTD)	Risk-to-benefit analysis of flour fortification with folic acid Environmental risk factors for folate-unresponsive NTD	Adverse effects of folic acid and dose-response relationship Pathogenesis of folate-unresponsive NTD; identification of relevant environmental agents	Inaccuracies and discrepancies in (a) NTD epidemiology (b) monitoring of folate status Effect of population variability (eg, socio-economic status, diet, genetic polymorphisms)	Moderate on the potential for primary prevention (adequate folate status already established as a factor reducing NTD incidence by approx 50%) Severe (both epistemic and sampling uncertainties) on the ability to assess the effects of primary prevention measures
Familial Mediterreanean Fever (FMF)	Genotype-phenotype correlation in FMF, in presence of numerous variants	Role of genetic variants in the severity and clinical manifestations of FMF. Model of inheritance Role of non-genetic factors	Inaccuracies and discrepancies in (a) diagnosis (b) characterization of genetic variants Effect of population variability	Moderate to severe (both epistemic and sampling uncertainties) on secondary and tertiary prevention, diagnosis and epidemiology. Epistemic uncertainties on role of genetic variants specifically affect the accuracy of genetic counselling
